# The c-Jun N-terminal kinase signaling pathway mediates chrysotile asbestos-induced alveolar epithelial cell apoptosis

**DOI:** 10.3892/mmr.2014.3119

**Published:** 2014-12-19

**Authors:** PENG LI, TIE LIU, DAVID W. KAMP, ZIYING LIN, YAHONG WANG, DONGHONG LI, LAWEI YANG, HUIJUAN HE, GANG LIU

**Affiliations:** 1 Clinical Research Center, Guangdong Medical College, Zhanjiang, Guangdong 524001, P.R. China; 2 Department of Hematology, The First Affiliated Hospital, Medical School of Xi’an Jiaotong University, Xi’an, Shaanxi 710061, P.R. China; 3 Department of Medicine, Division of Pulmonary and Critical Care Medicine, Jesse Brown Veterans Affairs Medical Center and Northwestern University Feinberg School of Medicine, Chicago, IL 60611, USA

**Keywords:** chrysotile asbestos, apoptosis, c-Jun N-terminal kinases (JNK), mitochondrial dysfunction

## Abstract

Exposure to chrysotile asbestos exposure is associated with an increased risk of mortality in combination with pulmonary diseases including lung cancer, mesothelioma and asbestosis. Multiple mechanisms by which chrysotile asbestos fibers induce pulmonary disease have been identified, however the role of apoptosis in human lung alveolar epithelial cells (AEC) has not yet been fully explored. Accumulating evidence implicates AEC apoptosis as a crucial event in the development of both idiopathic pulmonary fibrosis and asbestosis. The aim of the present study was to determine whether chrysotile asbestos induces mitochondria-regulated (intrinsic) AEC apoptosis and, if so, whether this induction occurs via the activation of mitogen-activated protein kinases (MAPK). Human A549 bronchoalveolar carcinoma-derived cells with alveolar epithelial type II-like features were used. The present study showed that chrysotile asbestos induced a dose- and time-dependent decrease in A549 cell viability, which was accompanied by the activation of the MAPK c-Jun N-terminal kinases (JNK), but not the MAPKs extracellular signal-regulated kinase 1/2 and p38. Chrysotile asbestos was also shown to induce intrinsic AEC apoptosis, as evidenced by the upregulation of the pro-apoptotic genes Bax and Bak, alongside the activation of caspase-9, poly (ADP-ribose) polymerase (PARP), and the release of cytochrome *c*. Furthermore, the specific JNK inhibitor SP600125 blocked chrysotile asbestos-induced JNK activation and subsequent apoptosis, as assessed by both caspase-9 cleavage and PARP activation. The results of the present study demonstrated that chrysotile asbestos induces intrinsic AEC apoptosis by a JNK-dependent mechanism, and suggests a potential novel target for the modulation of chrysotile asbestos-associated lung diseases.

## Introduction

Environmental or occupational exposure to asbestos fibers increase the risk for chronic respiratory diseases, including interstitial lung fibrosis (for example, asbestosis), lung cancer, and pleural malignant mesothelioma (1–4). Asbestos fibers are naturally found in rocks and soils and consist of six distinct types: Crocidolite, amosite, anthophyllite, tremolite, actinolite and chrysotile (4,5). Chrysotile asbestos is the sole serpentine type of asbestos (6,7), which has been widely used as an industrial material in China. The mechanisms of injury to the cells of the lung and pleura, resulting in lung diseases, following asbestos exposure have not yet been fully established, despite extensive investigations over several decades (1,2,4,7). All forms of asbestos, including chrysotile, are carcinogenic, and have been previously shown to promote iron-derived free radical formation *in vitro*, injure lung target cells, and induce asbestosis, lung cancer, and mesothelioma in humans (1,2,4,7). As previously reviewed, accumulating evidence firmly implicates alveolar epithelial cell (AEC) apoptosis as an important early event in the pathophysiology of idiopathic pulmonary fibrosis (IPF) and asbestosis (2,4). Previous studies have shown that asbestos-induced pulmonary toxicity is mediated in part by lung epithelial cell mitochondrial dysfunction, mitochondrial reactive oxygen species production, DNA damage, p53 activation and mitochondria-regulated apoptosis (2,4,7,8). Previous studies on lung epithelial cell mitochondria-regulated (intrinsic) apoptosis have focused solely on the effects of amphibole asbestos, including amosite and crocidolite, while the effects of chrysotile asbestos on lung epithelial cells is currently unknown (2–4,9–11).

Asbestos pulmonary toxicity, including apoptosis, occurs partly by the activation of epidermal growth factor receptor and other receptors, resulting in the activation of the mitogen-activated protein kinase (MAPK) pathway, which includes p38, c-Jun N-terminal kinase (JNK) and extracellular signal regulated kinase (ERK 1/2) (12,13). The MAPK p38 has been previously implicated in the chronic immune response elicited by asbestos in rat mesothelial cells (14). Furthermore, fibroblast growth factor-10 has been shown to decrease asbestos-induced DNA damage and apoptosis in AECs by modulating MAPK-ERK-dependent signaling that affects the mitochondria-regulated apoptosis pathway (15). Asbestos induces AEC plasticity through the MAPK-ERK signaling pathway (16). Protein kinase delta (PKCδ)-dependent mechanisms have been implicated in mediating AEC intrinsic apoptosis, in part through PKCδ phosphorylation of JNK which triggers pro-apoptotic Bcl-2-like protein 11 (Bim) expression (8). Whereas the ERK1/2-related anti-apoptotic pathways have been shown to be activated at lower asbestos concentrations as a survival response in tumor cells (8).

In the present study, it was hypothesized that chrysotile asbestos may induce AEC intrinsic apoptosis via the MAPK-JNK signaling pathway. To address this hypothesis, the effects of chrysotile asbestos on cell viability and DNA fragmentation were analyzed in human A549 bronchoalveolar carcinoma cells, with alveolar epithelial type II-like features. Chrysotile-induced A549 cell expression of activated JNK, ERK1/2, p38, B cell lymphoma-2 (Bcl-2) associated X protein (Bax), Bcl-2 homologous antagonist killer (Bak), cytochrome *c*, caspase-9 and poly (ADP-ribose) polymerase (PARP) proteins was also assessed by western blotting. Furthermore, the effects of the JNK inhibitor SP600125, on chrysotile asbestos-induced A549 cell apoptosis and JNK-PARP signaling were determined. The present study showed that chrysotile asbestos can induce intrinsic apoptosis in A549 cells through the JNK-dependent signaling pathway.

## Materials and methods

### Reagents

Dulbecco’s modified Eagle’s medium (DMEM) and fetal bovine serum (FBS) were obtained from Gibco-BRL (Carlsbad, CA, USA). Primary rabbit polyclonal antibodies against human phospho-JNK1/2, phospho-ERK1/2, phospho-p38, phospho-p53, JNK1/2, ERK1/2, p38, cytochrome *c*, PARP, Bax, Bak, and caspase-9 were purchased from Cell Signaling Technology, Inc. (Danvers, MA, USA), and an antibody against β-actin was purchased from Santa Cruz Biotechnology Inc. (Santa Cruz, CA, USA). The antibodies were used at a 1:2,000 dilution. The JNK inhibitor SP600125, was purchased from Calbiochem^®^ (Merck Millipore, La Jolla, CA, USA). Polyvinylidene difluoride membrane (PVDF) was purchased from Millipore (Billerica, MA, USA). Cellular DNA Fragmentation ELISA kits were purchased from Roche (Basel, Switzerland), the DeadEnd™ Fluorometric terminal deoxynucleotidyl transferase-mediated dUTP nick-end labeling (TUNEL) system was purchased from Promega Corporation (Madison, WI, USA), and the PrimeScript Reverse Transcription (RT) Enzyme Mix kits and SYBR^®^ Green polymerase chain reaction (PCR) reagent were purchased from Takara Biotechnology Co., Ltd. (Osaka, Japan). All the reagents used throughout the study were of analytical or cell culture grade purity. Chrysotile asbestos was obtained from Mangya Moutain (Qinghai Province, China).

### Pretreatment of chrysotile asbestos

Chrysotile asbestos fibers used in the following experiments were mined from Qinghai, China. The chrysotile asbestos used in the treatments had an average length of 7.8 μm, and an average diameter of 0.2 μm, which was confirmed using transmission electron microscopy. The fibers were prepared as described by previous methods (17). Briefly, fiber samples were weighed and crushed into an ultrafine powder, using a mechanical crusher (Xulang Machinery Equipment Co., Ltd, Guangzhou, China). Following ultra-sonication at 20 kHz for 10 min using a bath-type sonicator (Q700; QSonica LLC, Newtown, CT, USA), the mixtures were centrifuged at 2,000 × g for 10 min, the supernatants were removed and the pellets were washed with 2 ml distilled water. Each chrysotile asbestos sample was then re-suspended in phosphate-buffered saline (PBS) for the cell treatment. A stock solution of the fibers (5 mg/ml) was sterilized by autoclaving and mixed to ensure a uniform suspension prior to dilution with tissue culture medium, ready for the cell treatment.

### Cell culture

A549 human bronchoalveolar carcinoma-derived cells, with some features characteristic of alveolar epithelial type II cells, were obtained from the American Type Culture Collection (Manassas, VA, USA). The cells were cultured in a humidified chamber containing 5% CO_2_ at 37°C and maintained in DMEM supplemented with 10% FBS and antibiotics (100 U/ml penicillin and 100 g/ml streptomycin). For the experiments, A549 cells were plated in 35 mm diameter dishes (2×10^5^ cells/dish). Following 24 h in culture, the medium was refreshed with 4 ml medium containing chrysotile fibers at the indicated final concentrations.

### Measurement of cell viability by trypan blue exclusion method

The trypan blue exclusion method is a classic cell procedure used for assessing cell viability. The cells were divided and cultured in six-well plates (1×10^5^ cells/well) for 24 h, followed by treatment with chrysotile asbestos at various concentrations for 24 h. The cells were harvested and centrifuged (5810R; Eppendorf, Hamburg, Germany) to remove the medium. The cells were then washed in PBS three times and resuspended, resulting in a suspension of 1×10^6^ cells/ml. The suspension was mixed with 0.4% trypan blue dye (Sigma-Aldrich, St. Louis, MO, USA) for 5 min at 25°C. The unstained (viable) and stained (non-viable) cells were counted using a hemacytometer (Neubauer Improved; Marienfeld, Lauda-Königshofen, Germany) within 5 min in four microscope fields, at magnification ×40, per well (>100 cells/field).

### Detection of apoptotic cells

Experiments were performed using a cellular DNA fragmentation ELISA kit according to the manufacturer’s instructions (Roche). A549 cells were labeled with 10 μM bromodeoxyuridine (BrdU; Roche) at 1×10^5^ cells/ml. BrdU-labeled cells (1×10^4^) in 100 μl were treated with varying concentrations (100, 150, 200 μg/cm^2^) of cell extract for a period of 4 h. Following treatment, the cells were lysed with lysis buffer (20 mM Tris, pH 7.6; 1% Triton X-100; 137 mM NaCl; 2 mM EDTA; 1 mM Na_3_O_4_V; 10 mM NaF; 1 mM DTT; 1 mM phenylmethylsulfonyl fluoride; 10 μg/ml leupeptin and 10 μg/ml aprotinin) for 30 min at 25°C, and the supernatants containing apoptotic fragments were obtained following centrifugation at 1,500 × g for 10 min. The recovered samples (100 μl) were transferred onto anti-DNA-coated 96-well flat-bottom microplates (Corning Inc., Corning, NY, USA). The plates were incubated for 90 min at 15–25°C, and the wells were washed three times with washing buffer, which was provided in the DNA fragmentation ELISA kit, for 2–3 min per wash. The DNA bound to the coated microplates was denatured by nuclease treatment (exonuclease III solution, 37°C for 30 min; Takara Biotechnology Co., Ltd), followed by the addition of 100 μl anti-BrdU-peroxidase (POD; Roche) conjugate solution. The plates were incubated for an additional 90 min and were washed again with washing buffer. Following washing, 100 μl of the substrate solution was added, and the plates were shaken until color development was deemed sufficient. The absorbance was measured at 450 nm following the addition of 25 μl of stop solution (Roche).

### TUNEL assay

DNA damage was assessed by TUNEL assay using an *in situ* Cell Death Detection kit with fluorescein-dUTP as a label, according to the manufacturer’s instructions (Promega Corporation). A549 cells were plated onto confocal petri dishes and grown to confluence over 24 h in DMEM supplemented with 10% FBS, followed by treatment with chrysotile asbestos for 24 h. Following incubation, the culture medium was removed and the cells were washed three times in PBS. The cells were then fixed with 4% paraformaldehyde (Sigma-Aldrich), permeabilized with 0.1% Triton X-100 (Sigma-Aldrich), and incubated in the dark at 37°C for 1 h in a TUNEL reaction mixture containing 50 μl of a mixture of terminal deoxynucleotidyl transferase and dUTP. DAPI was added at 25°C for 10 min as a non-specific stain of the cellular nuclei. Five fields per dish of cells were randomly analyzed using a Leica TCS SP5 II confocal microscope (Leica, Wetzlar, Germany)(>100cells/field). Each DAPI-stained cell was categorized as apoptotic if green nuclear fluorescence was observed, or normal if no green fluorescence was observed.

### Western blot analysis

The treated cells were rinsed with ice-cold PBS and incubated with radioimmunoprecipitation assay lysis buffer containing 50 mM Tris-HCl (pH 7.4), 150 mM NaCl, 1% Triton-X 100, 1% sodium deoxycholate, 0.1% sodium dodecyl sulfate (SDS), 1 mM ethylenediaminetetraacetic acid, 1 mM sodium fluoride, 1 mM phenylmethanesulfonyl fluoride, 10 μg/ml aprotinin, 1 μg/ml leupeptin, and 1 μg/ml pepstatin for 20 min. The cell lysates were then centrifuged at 12,000 × g for 15 min, and protein concentrations were determined using the Bicinchoninic Acid Protein Assay kit (Beyotime, Jiangsu, China). Total cell protein (20 μg/lane) was separated by 10 or 12% SDS-PAGE followed by transfer to PVDF membranes. The membranes were blocked for 1 h in Tris-buffered saline containing 0.05% Tween-20 (TBST), with 5% nonfat dry milk. The membranes were then incubated with rabbit polyclonal antibodies against phospho-JNK1/2, phospho-ERK1/2, phospho-p38, phospho-p53, JNK1/2, ERK1/2, p38, cytochrome *c*, Bax, Bak, caspase-9, PARP or β-actin overnight at 4°C. Following primary antibody incubation the membranes were washed with TBST and incubated for 1 h with goat anti-rabbit immunoglobulin G-conjugated horseradish peroxidase-conjugated secondary antibody. The antibody-reactive bands were revealed using an enhanced chemiluminescence reagent (GE Healthcare, Little Chalfont, UK) and exposed to radiographic film.

### Real-time quantitative polymerase chain reaction (PCR) (qPCR) analysis

The cells were treated with chrysotile asbestos for the indicated times, followed by extraction of total RNA using TRIzol reagent (Invitrogen Life Technologies, Carlsbad, CA, USA) according to the manufacturer’s instructions. Total RNA (1 μg) was reverse-transcribed into cDNA using PrimeScript RT Enzyme mix at 37°C for 15 min, followed by an 85°C incubation for 5 s. Specific primers for real-time qPCR are detailed in Table 1. GAPDH was used as an internal control.

Amplification was performed in a 20 μl total reaction volume using real-time SYBR^®^ Green PCR reagent in a LightCycler^®^ 480II Real-Time thermal cycler (Roche). The cycling conditions were as follows: 95°C for 30 s, followed by 40 cycles of 95°C for 5 s and 60°C for 20 s. Melting-curve analysis was performed for each primer set, to ensure that no primer dimers or nonspecific amplification was present under the optimized cycling conditions. The fold difference in mRNA expression was determined using the relative quantification method, with normalization to GAPDH mRNA, by comparing the relative cycle threshold (Ct) changes between the control and the experimental samples. The fold change was calculated from the mean of the control group for each individual sample, including individual control samples, to assess variability within the groups.

### Statistical analysis

The data are presented as the means ± standard deviation. Statistical significance was evaluated using a Student’s t-test. When more than one group was compared with a control, the significance was evaluated according to a one-way analysis of variance, and a Duncan’s *post hoc* test was applied to identify group differences. A P<0.05 was considered to indicate a statistically significant difference. The statistical package SPSS, version 11.0 for Windows (SPSS Inc., Chicago, IL, USA) was used for statistical analyses.

## Results

### Chrysotile asbestos decreases cell viability and induces apoptosis in A549 cells

**Figure 1:**
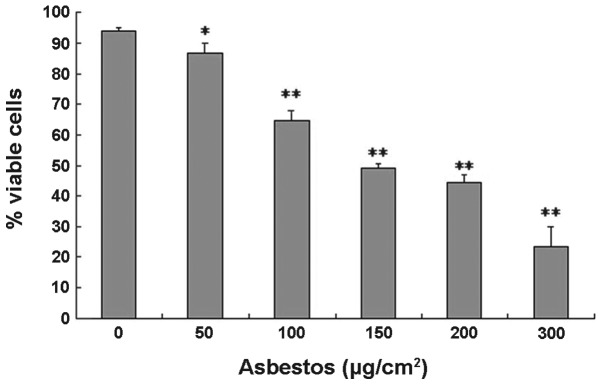
Effects of increasing concentrations of chrysotile asbestos on A549 human bronchoalveolar carcinoma cell viability. The viability of the cells was determined by trypan blue exclusion assay using a hemacytometer. The cell viability was shown to be significantly different between the chrysotile asbestos-treated samples and controls. The cell viability rates are expressed as percentages of the control values. The values represent the means ± standard deviation of three independent experiments. The statistical significance of the results was analyzed by the Student’s t-test, ^*^P<0.05, ^**^P<0.01, vs control.

**Figure 2:**
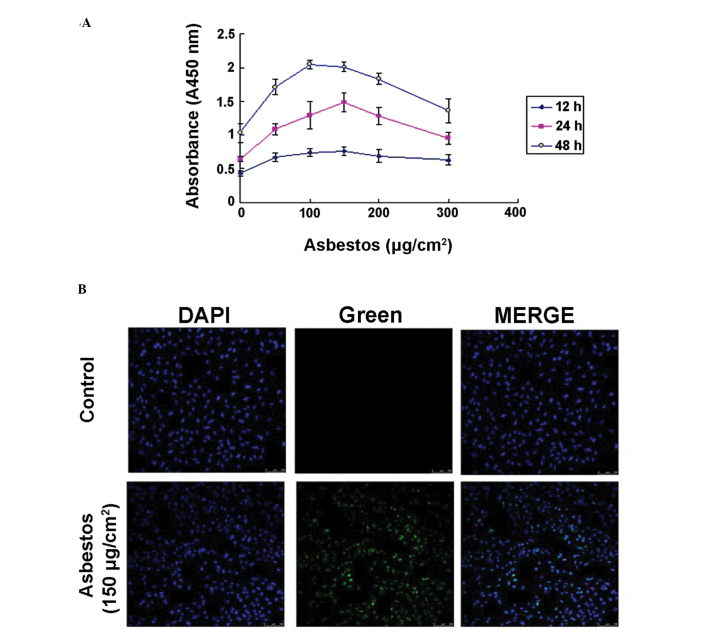
Induction of apoptosis by chrysotile asbestos in A549 human bronchoalveolar carcinoma cells. (A) A dose and time-dependent increase in apoptosis in A549 cells was observed following chrysotile asbestos treatment, as determined by a cellular DNA fragmentation ELISA. The data represent the means ± standard deviation of three independent experiments. (B) A terminal deoxynucleotidyl transferase-mediated dUTP nick end labeling assay of asbestos-induced A549 cells was performed following an incubation with 150 μg/cm^2^ chrysotile asbestos for 24 h. The apoptotic nuclei stained green, whereas all nuclei stained blue (DAPI). Magnification, ×400. Results are representative of three independent experiments. H, hours; nm, nanometers.

To examine chrysotile asbestos-induced cytotoxicity on A549 cells, the effects of chrysotile asbestos on cell survival were determined. The cells were treated for 24 h with increasing concentrations of chrysotile asbestos, ranging from 50–300 μg/cm^2^. As the concentration of chrysotile asbestos increased there was a significant decrease in the number of viable cells, as assessed using the trypan blue exclusion assay. For the control untreated A549 cells, the viability was 93.87%, and for the cells treated with chrysotile asbestos (50, 100, 150, 200, 300 μg/cm^2^), viability was 86.5, 64.93, 49.1, 44.43, and 23.63%, respectively (Fig. 1). To further investigate whether chrysotile asbestos induces apoptosis in A549 cells, the levels of cellular DNA fragmentation, a hallmark of apoptotic cell death, were assessed. The results indicated that chrysotile asbestos induced DNA fragmentation in a dose- and time-dependent manner, with the levels peaking at ~100–150 μg/cm^2^ chrysotile asbestos (Fig. 2A). To verify these findings, DNA cleavage was assayed by TUNEL staining, following 24 h exposure of A549 cells to chrysotile asbestos. Chrysotile asbestos led to an increased intensity of green fluorescence in the nuclear region of the cells (Fig. 2B), further confirming that chrysotile asbestos may induce apoptotic cell death.

### Chrysotile asbestos induces apoptosis in A549 cells, via the JNK-MAPK signaling pathway

**Figure 3:**
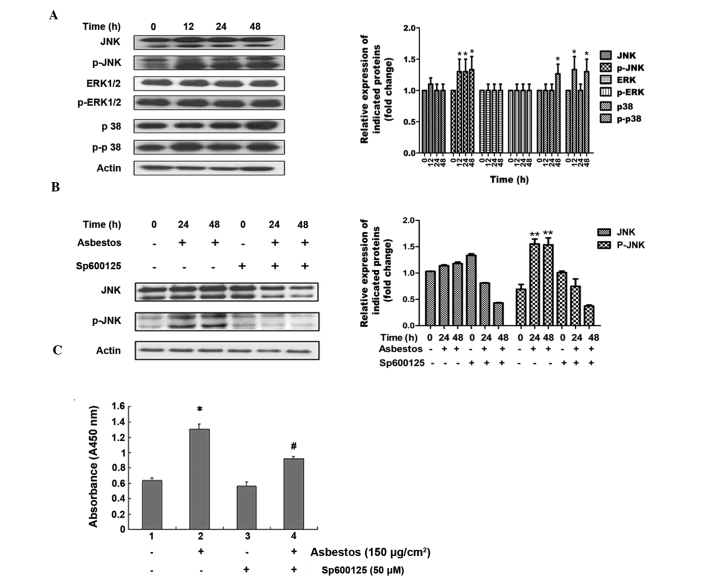
Effects of chrysotile asbestos on mitogen-activated protein kinase (MAPK) activation in A549 human bronchoalveolar carcinoma cells. (A) A549 cells were left untreated or were treated with chrysotile asbestos (150 μg/cm^2^) for 12, 24 or 48 h, and the phosphorylation of c-Jun N-terminal kinase (JNK)-1/2, extracellular signal-regulated kinase (ERK)-1/2 and p38-MAPK were assessed by western blotting. The results are representative of three independent experiments. (B) A549 cells were treated with chrysotile asbestos (150 μg/cm^2^) for 24 or 48 h in the absence or presence of the JNK specific inhibitor SP600125 (50 μM for 5 h prior to chrysotile asbestos treatment), and the phosphorylation of JNK protein was examined by western blotting. The results are representative of three independent experiments. (C) A549 cells were pretreated with 50 μM SP600125 for 5 h prior to 150 μg/cm^2^ asbestos treatment. Following 24 h incubation, the apoptotic fragmentation was assessed with a cellular DNA fragmentation ELISA. The values shown represent the means ± standard deviation from three independent experiments. The statistical significance of the results was analyzed by a Student’s t-test, ^*^P<0.05 vs. untreated control; ^#^P<0.05 vs. asbestos-treated cells. H, hours; JNK, c-Jun N-terminal kinase; ERK, extracellular signal-regulated kinase; nm, nanometers.

MAPKs have an important role in certain apoptotic signaling pathways. Therefore, the possible role of MAPKs in chrysotile asbestos-induced A549 cell apoptosis was determined. As compared with the control untreated cells, the chrysotile asbestos-treated A549 cells had significantly increased levels of phosphorylated (activated) JNK1/2 protein, but not of phosphorylated ERK1/2 or p38 (Fig. 3A). The effects of chrysotile asbestos on the JNK1/2 pathway could be reversed by pre-treatment of the cells with the JNK inhibitor SP600125 (Fig. 3B). A marked increase in the relative expression of JNK mRNA was observed following chrysotile asbestos exposure (150 μg/cm^2^) (Fig. 3C). To investigate the role of JNK in asbestos-induced A549 cell death, apoptosis levels were assessed following treatment with SP600125 (Fig. 3D). Chrysotile asbestos-induced apoptosis was partially decreased by treatment with SP600125. These results further suggest that JNK has a role in chrysotile asbestos-induced apoptosis of A549 cells.

### Chrysotile asbestos induces intrinsic apoptosis in A549 cells, and inhibition of JNK is protective

**Figure 4:**
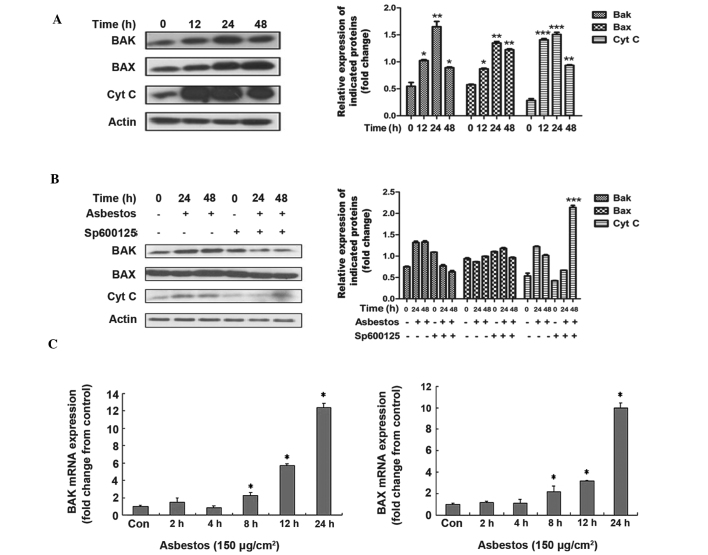
Analysis of mitochondrial dysfunction in chrysotile asbestos-treated A549 human bronchoalveolar carcinoma cells. (A) The cells were either left untreated or were treated with chrysotile asbestos (150 μg/cm^2^) for 12, 24 or 48 h, and the expression of pro-apoptotic genes Bak and Bax, and cytochrome c was assessed by western blotting. The results are representative of three independent experiments. (B) c-Jun N-terminal kinase (JNK) inhibitor SP600125 (50 μM for 5 h) was added to A549 cells prior to treatment with chrysotile asbestos (150 μg/cm^2^ for 0, 24 or 48 h), Bax/Bak, and cytochrome c levels were examined by western blotting. The results are representative of three independent experiments. (C) The mRNA levels of Bak and Bax were assessed by quantitative polymerase chain reaction following the treatment of A549 cells with chrysotile asbestos (150 μg/cm^2^ for 0, 2, 4, 8, 12 or 24h). The data represent the means ± standard deviation from three independent experiments. The statistical significance of the results was analyzed by a Student’s t-test, ^*^P<0.05 vs. untreated control.Bax, B cell lymphoma-2 (Bcl-2) associated X protein; Bak, Bcl-2 homolohous antagonist killer; Cyt C, cytochrome c; h, hours; Con, control.

The Bcl-2 family of pro- and anti-apoptotic proteins modulate the stability of the mitochondrial membrane, which determines whether or not the intrinsic apoptosis pathway is activated (18). To investigate whether the apoptotic response to chrysotile asbestos is mediated through this pathway, levels of pro-apoptotic Bax and Bak were assessed, using western blotting and qPCR. As shown in Fig. 4A, chrysotile asbestos treatment induced a time-dependent increase in the levels of Bax and Bak protein. As confirmation, the release of cytochrome *c* from the mitochondria into the cytosol was also shown to be markedly increased in response to chrysotile asbestos treatment. The increases in Bax/Bak and cytochrome *c* expression levels were reversed by pretreatment with SP600125, suggesting that JNK signaling regulates the effects on mitochondrial stability by chrysotile asbestos (Fig. 4B). Furthermore, 150 μg/cm^2^ chrysotile asbestos induced an increase in the mRNA expression of Bax/Bak (Fig. 4C and D), thus suggesting that the regulation may occur at the transcriptional level.

### Chrysotile asbestos induces A549 cell cleaved caspase-9 and PARP, and inhibition of JNK is protective

**Figure 5:**
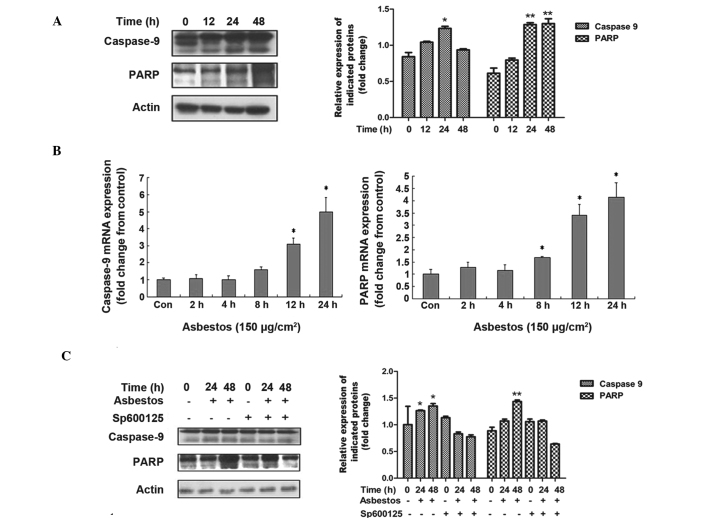
Chrysotile asbestos induces caspase activation and cleavage of poly (ADP-ribose) polymerase (PARP) in A549 human bronchoalveolar carcinoma cells. (A) The cells were treated with chrysotile asbestos (150 μg/cm^2^) for 12, 24 or 48 h, and the cleavage of caspase-9 and PARP was assessed by western blotting. The results are representative of three independent experiments. (B) The cells were treated with chrysotile asbestos (150 μg/cm^2^) for 0, 2, 4, 8, 12 or 24 h, and caspase-9 and PARP mRNA expression was detected by quantitative polymerase chain reaction. The values shown represent the means ± standard deviation from three independent experiments. (C) A549 cells were treated with chrysotile asbestos (150 μg/cm^2^) for 0, 24 or 48 h in the absence or presence of c-Jun N-terminal kinase (JNK) inhibitor SP600125 (50 μM for 5 h), and caspase-9 and PARP were then examined by western blotting. The results are representative of three independent experiments. The statistical significance of the results was analyzed by a Student’s t-test (^*^p<0.05 vs. untreated control). Con, control;h, hours.

Caspase-9 and PARP, a substrate of caspase-3, represent two additional cellular proteins that are known to be activated during apoptotic signaling (19). To determine whether these proteins are activated by chrysotile asbestos, the relative protein and mRNA expression levels were determined following treatment. As shown in Fig. 5A and B, a marked increase in protein and mRNA expression is observed for both caspase-9 and PARP, following chrysotile asbestos treatment. Furthermore, caspase-9 and PARP activation were blocked by the JNK inhibitor SP600125, verifying the role for JNK in mediating chrysotile asbestos-induced cell death (Fig. 5C).

## Discussion

Asbestos-related pulmonary diseases remain an important long-term health concern world-wide. This is in part due to the vast amount of fibers mined and used for numerous industrial purposes and the long lag phase between exposure to the fibers and initiation of disease, which can be between 20–40 years (20). The U.S. Environmental Protection Agency has banned the production and use of all types of asbestos (20). However, in some developing countries, including China, asbestos fibers, especially chrysotile, are still widely used as a construction material. Although there are numerous pathophysiological mechanisms accounting for asbestos-induced pulmonary toxicity, AEC apoptosis is widely implicated but by mechanisms which are not yet fully understood (1,2,4,7). Furthermore, the role of chrysotile asbestos in mediating human AEC apoptosis is not well characterized. The present study showed that chrysotile asbestos induced intrinsic apoptosis in human A549 lung epithelial cells, in a dose- and time-dependent manner, as assessed by cell viability, DNA fragmentation, and expression of Bax-Bak, cleaved caspase-9 and PARP activation. Furthermore, it was determined that chrysotile asbestos-induced A549 cell intrinsic apoptosis was mediated by JNK activation. Overall, these results suggest that chrysotile-induced AEC JNK activation is a novel signaling pathway which may be relevant in the pathophysiology of asbestos-related pulmonary diseases.

Apoptosis, also known as programmed cell death, is an important mechanism by which cells with DNA damage are eliminated without the elicitation of an inflammatory response (21). Apoptotic cells are characterized by nuclear chromatid condensation, endonuclease activation resulting in DNA fragmentation, translocation of phosphatidylserine to the outer plasma membrane, and the generation of double-stranded DNA breaks (22–24). Asbestos has been shown to trigger apoptosis in all relevant lung target cells (25). Previous studies have shown that low levels of asbestos (<0.5 μg/cm^2^) promote the entry of cells into the S-phase of the cell cycle without inducing apoptosis, whereas higher levels of asbestos (1.0–5.0 μg/cm^2^) impede entry into the S-phase and induce apoptosis (25–28). The results of the present study indicated that chrysotile asbestos induced the greatest extent of apoptosis in A549 cells at a concentration of 150 μg/cm^2^ for 48 h, likely due to the lower cytotoxicity of chrysotile asbestos as compared with other types of asbestos (20). A previous study showed that asbestos induces protein kinase C δ (PKCδ)-dependent protein kinase D (PKD) phosphorylation in lung epithelial cells (8). PKCδ-dependent PKD phosphorylation by asbestos is causally linked to a cellular pathway that involves the phosphorylation of both ERK1/2 and JNK1/2, which have opposing roles in the apoptotic response induced by asbestos (8). Furthermore, numerous studies have previously shown that AEC apoptosis, via caspase-3 and -9 activation, mediates asbestos-induced lung injury (11, 29, 30). The results of the present study showed that, in high doses, chrysotile asbestos triggers apoptosis of A549 cells by inducing the release of cytochrome *c*. This release was shown to be accompanied by a marked increase in the activation of Bax/Bak and caspase-9. Furthermore, the exposure of A549 cells to chrysotile asbestos resulted in a significant increase in the relative expression levels of Bax/Bak and Caspase-9 mRNA. These results indicate that chrysotile asbestos induces A549 cell apoptosis through the mitochondria-dependent pathway.

The MAPKs (p38, ERK, and JNK) are activated in response to various cellular stressors or stimuli, including oxidative stress, lipopolysaccharide and tumor necrosis factor-α (31–33). Generally, the JNK and p38 MAPK pathways are considered as “stress-activated protein kinases”, which have an essential role in inflammation and apoptosis (34). The ERK pathway has been shown to be important in cellular differentiation and proliferation, as well as in cell survival (34). To evaluate the involvement of the MAPK signaling pathways in chrysotile asbestos-induced A549 cell apoptosis, the expression of MAPKs, following treatment with chrysotile asbestos, was determined in the present study (12, 24 and 48h). Chrysotile asbestos led to a significant time-dependent activation of the JNK protein, whereas there were no significant changes in the activation of p38 and ERK (Fig. 3). JNK has previously been shown to phosphorylate mitochondrial membrane proteins, such as the Bcl-2 family members, activating their apoptotic function (35,36). Furthermore, caspases transduce the signals of most apoptosis-inducing factors (37), and PARP is one of the main cleavage targets of caspase-3 and a main effector in cell apoptosis (19). Therefore, to determine the molecular mechanisms of chrysotile asbestos-induced apoptosis in A549 cells, the expression levels of Bax/Bak, caspase-9 and PARP were determined, following the treatment of A549 cells with chrysotile asbestos. Treatment with chrysotile asbestos increased the expression of each of these proteins at different time points. Moreover, the JNK-specific inhibitor SP600125 substantially inhibited chrysotile asbestos-induced activation. Apoptosis was also significantly decreased by SP600125 pre-treatment (Fig. 4 and 5), suggesting that the JNK signaling pathway is functionally involved in chrysotile asbestos-induced A549 cell apoptosis through these downstream effectors.

**Figure 6:**
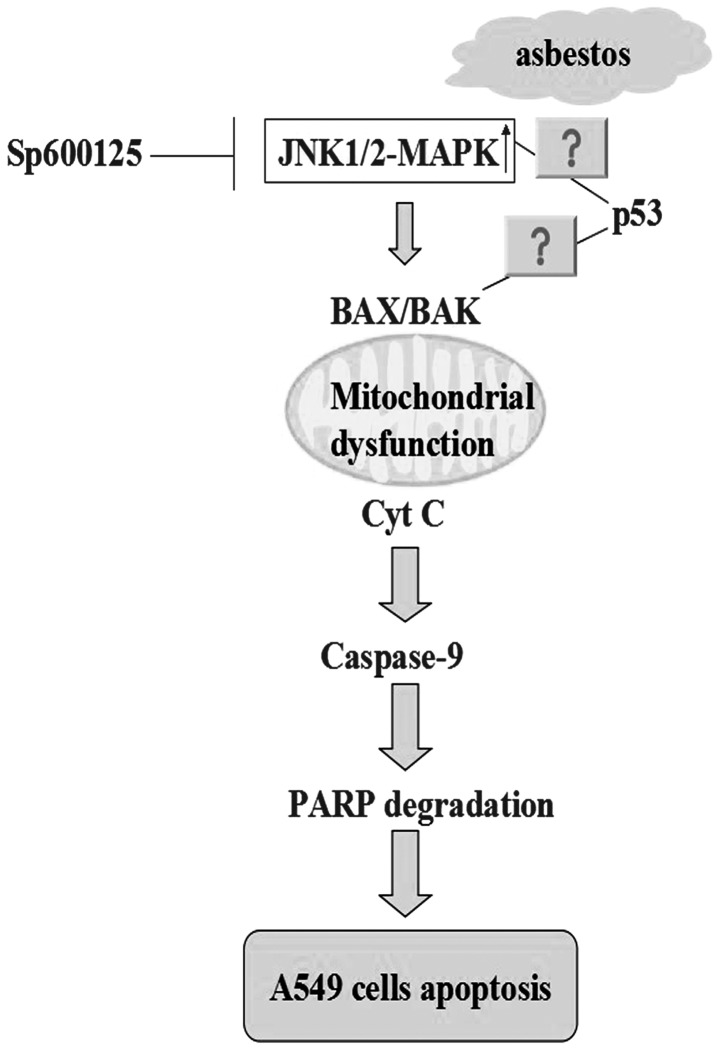
A schematic representation of the molecular mechanism proposed for the induction of apoptosis by chrysotile asbestos in A549 human bronchoalveolar carcinoma cells. The results from the present study support a pathway by which chrysotile asbestos induces apoptosis via the activation of c-Jun N-terminal kinase (JNK), resulting in mitochondrial disruption, the release of cytochrome c and finally the activation of a cascade of caspases. JNK, c-Jun N-terminal kinase; MAPK, mitogen-activated protein kinases; BAX, B cell lymphoma-2 (Bcl-2) associated X protein; BAK, Bcl-2 homologous antagonist killer; Cyt C, cytochrome c; PARP, poly (ADP-ribose) polymerase; Sp00125, JNK inhibitor.

In conclusion, the results of the present study revealed that chrysotile asbestos significantly decreased the viability of human A549 cells. Furthermore, chrysotile asbestos triggered mitochondrial dysfunction and intrinsic apoptotic cascades through the activation of JNK1/2 phosphorylation, which was shown to be reversed by the specific JNK inhibitor SP600125. A hypothetical model illustrating the role of the JNK signaling pathway in chrysotile asbestos-induced apoptosis is shown in Fig 6. The precise molecular mechanisms by which chrysotile-induced JNK activation triggers AEC intrinsic apoptosis, as well as the *in vivo* relevance of the present *in vitro* findings, requires further study. Chrysotile asbestos-induced intrinsic AEC apoptosis through a JNK-dependent mechanism may be a novel target for the modulation of chrysotile asbestos-related lung diseases.

## Figures and Tables

**Table I:** Primer seqences used for quantitative polymerase chain reaction.

Gene	Primer sequence (5′-3′)
Bax	F: AAGCTGAGCGAGTGTCTCAAG R: CAAAGTAGAAAAGGGCGACAAC
Bak	F: AGGACACAGAGGAGGTTTTCC R: ATAGCGTCGGTTGATGTCGT
JNK	F: CTTTGCCAAGTGATTCAGATGGA R: TTACTGGGCTTTAAGTCCCGATG
Caspase-9	F: CTAACAGGCAAGCAGCAAAGT R: GACATCACCAAATCCTCCAGA
PARP	F: AGGACGACAAGGAAAACAGGTA R: CATAGTCAATCTCCAGGGGGTA
GAPDH	F: AGAAGGCTGGGGCTCATTTG R: AGGGGCCATCCACAGTCTTC

F, forward; R, reverse.
